# Metasurface‐Embedded Contact Lenses for Holographic Light Projection

**DOI:** 10.1002/advs.202407045

**Published:** 2024-08-09

**Authors:** Jiwoo Ko, Gyeongtae Kim, Inki Kim, Soon Hyoung Hwang, Sohee Jeon, Junseong Ahn, Yongrok Jeong, Ji‐Hwan Ha, Hyeonsu Heo, Jun‐Ho Jeong, Inkyu Park, Junsuk Rho

**Affiliations:** ^1^ Department of Mechanical Engineering Korea Advanced Institute of Science and Technology (KAIST) Daejeon 34141 South Korea; ^2^ Department of Nano Manufacturing Technology Korea Institute of Machinery and Materials (KIMM) Daejeon 34103 South Korea; ^3^ Department of Mechanical Engineering Pohang University of Science and Technology (POSTECH) Pohang 37673 Republic of Korea; ^4^ Department of Biophysics Institute of Quantum Biophysics Sungkyunkwan University Suwon 16419 Republic of Korea; ^5^ Department of Intelligent Precision Healthcare Convergence Sungkyunkwan University Suwon 16419 Republic of Korea; ^6^ Department of Electro‐Mechanical Systems Engineering Korea University Sejong 30019 Republic of Korea; ^7^ Radioisotope Research Division Korea Atomic Energy Research Institute Daejeon 34057 Republic of Korea; ^8^ Department of Chemical Engineering Pohang University of Science and Technology (POSTECH) Pohang 37673 Republic of Korea; ^9^ Department of Electrical Engineering Pohang University of Science and Technology (POSTECH) Pohang 37673 Republic of Korea; ^10^ POSCO‐POSTECH‐RIST Convergence Research Center for Flat Optics and Metaphotonics Pohang 37673 Republic of Korea; ^11^ National Institute of Nanomaterials Technology (NINT) Pohang 37673 Republic of Korea

**Keywords:** biocompatibility, hyaluronic acid, metasurface, nanotransfer, smart contact lens

## Abstract

Contact lenses have been instrumental in vision correction and are expected to be utilized in augmented reality (AR) displays through the integration of electronic and optical components. In optics, metasurfaces, an array of sub‐wavelength nanostructures, have offered optical multifunctionality in an ultra‐compact form factor, facilitating integration into various imaging, and display systems. However, transferring metasurfaces onto contact lenses remains challenging due to the non‐biocompatible materials of extant imprinting methods and the structural instability caused by the swelling and shrinking of the wetted surface. Here, a biocompatible method is presented to transfer metasurfaces onto contact lenses using hyaluronic acid (HA) as a soft mold and to allow for holographic light projection. A high‐efficiency metahologram is obtained with an all‐metallic 3D meta‐atom enhanced by the anisotropy of a rectangular structure, and a reflective background metal layer. A corrugated metal layer on the HA mold is supported with a SiO_2_ capping layer, to avoid unwanted wrinkles and to ensure structural stability when transferred to the surface of pliable and wettable contact lenses. Biocompatible method of transferring metasurfaces onto contact lenses promises the integration of diverse optical components, including holograms, lenses, gratings and more, to advance the visual experience for AR displays and human‐computer interfaces.

## Graphical abstract

The metasurface‐embedded contact lens for holographic light projection. Using biocompatible hyaluronic acid (HA) material, a metasurface with 3D metallic nanostructures is integrated into contact lenses. The metallic nanostructures are encapsulated with a SiO_2_ capping layer, which significantly enhances process stability. Under the circularly polarized light incidence, the reflected light from the all‐metallic metasurface generates a desired holographic image.

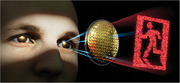

## 1 Introduction

Metasurfaces are composed of 2D arrays of subwavelength nanostructures, referred to as meta‐atoms. They have received significant attention for their ability to manipulate multiple properties of light, such as amplitude, phase, polarization, or orbital angular momentum, with high precision.^[^ ^1^, ^2^, ^3^, ^4^, ^5^ ^]^ Metasurfaces have been applied in various optical and electromagnetic domains, such as metalenses,^[^ ^6^, ^7^, ^8^ ^]^ metahologram,^[^ ^9^, ^10^, ^11^, ^12^, ^13^, ^14^ ^]^ and vortex beam generation,^[^ ^15^, ^16^ ^]^ enabling ultra‐compact imaging and display devices.^[^ ^17^, ^18^, ^19^ ^]^ Particularly, metahologram can reduce the physical size of devices and expand the field‐of‐view (FoV) up to 180°.^[^ ^20^ ^]^ Current near‐eye displays (NEDs) using head‐mounts or smart glasses still suffer from small FoV, eyebox and bulkiness of the entire system. To address these problems, previous methods have applied metasurfaces as eyepieces in glasses for both augmented and virtual reality (AR/VR) NEDs.^[^ ^17^, ^21^, ^22^ ^]^ However, the diameter of the metasurfaces has to be larger than 2 cm to achieve an FoV of 90°, which implies that the fabrication difficulty would be increased when the larger FoV is required.^[^ ^17^ ^]^ In contrast, reducing the eye‐relief, the distance between the human eye and the eyepiece, can increase the FoV without large diameter metasurfaces. However, the existing NEDs paired with head mounts or glasses face a fundamental constraint in reducing the size of eye relief.

Contact lenses, worn directly on the eye, reduce eye relief to near zero and present an ideal form factor for the most compact NEDs. These lenses can integrate functional optical and electronic components, such as displays or sensors, to present information and collect data. The integration of metasurfaces into contact lenses, replacing traditional display components or eyepieces, requires intricate nanostructures for higher‐resolution imaging, while ensuring the use of biocompatible materials for transfer. Prior research in the realm of smart contact lenses has employed various techniques, including imprinting patterns onto a transparent film for subsequent integration onto a soft lens,^[^ ^23^, ^24^ ^]^ utilizing laser interference,^[^ ^25^ ^]^ employing near‐field sub‐diffraction photolithography,^[^ ^26^ ^]^ and traditional photolithography methods.^[^ ^27^, ^28^ ^]^ Previous studies in this field have seen limitations; they have often relied on hazardous chemicals^[^ ^23^, ^24^, ^25^, ^26^, ^27^, ^28^ ^]^ or restricted themselves to specific nanopattern designs like nanolines or dot patterns.^[^ ^29^ ^]^ Nanoline patterns offer robustness in transfer due to their continuous structure, and dot patterns, being consistently geometric, are less impacted by rotational shifts during transfer. However, these approaches might not be suitable for metasurfaces with more complex nanostructures, indicating a need for more advanced embedding processes. Furthermore, the flexible and absorbent nature of contact lens materials poses challenges in maintaining precise nanostructure alignment.

In this study, we employed a biocompatible process to integrate a metasurface with 3D metallic nanostructures onto contact lenses. The metasurface, comprising 3D corrugated metallic nanostructures with varying rotation angles, facilitates holographic image generation through high reflectance from a background gold film, and helicity conversion from anisotropic rectangular meta‐atoms. Our 3D corrugated metallic nanostructures are encapsulated with a SiO_2_ capping layer, which provides robust anchoring, acts as a waveguide, and significantly enhances both process stability and overall performance. The visualization of the meta‐hologram has substantiated the feasibility of producing smart contact lenses with the most diminutive form factor. This approach demonstrates high hologram efficiency and represents a significant advancement in wearable optics. Our work is among the first to successfully integrate metasurfaces into contact lenses, paving the way for future applications in NEDs.

## 2 Results and Discussion

### 2.1 Design of All‐Metallic Geometric Metasurface

**Figure 1:**
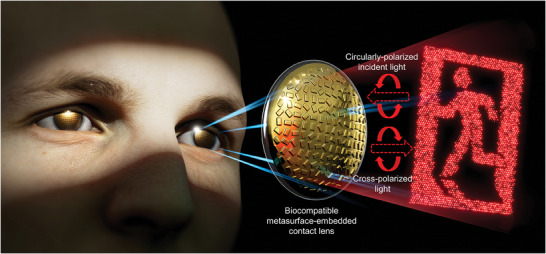
Schematic representation of metasurface‐embedded contact lens for holographic light projection. Under the circularly‐polarized light incidence, the reflected light from all‐metallic metasurface generates desired holographic image.

**Figure 2:**
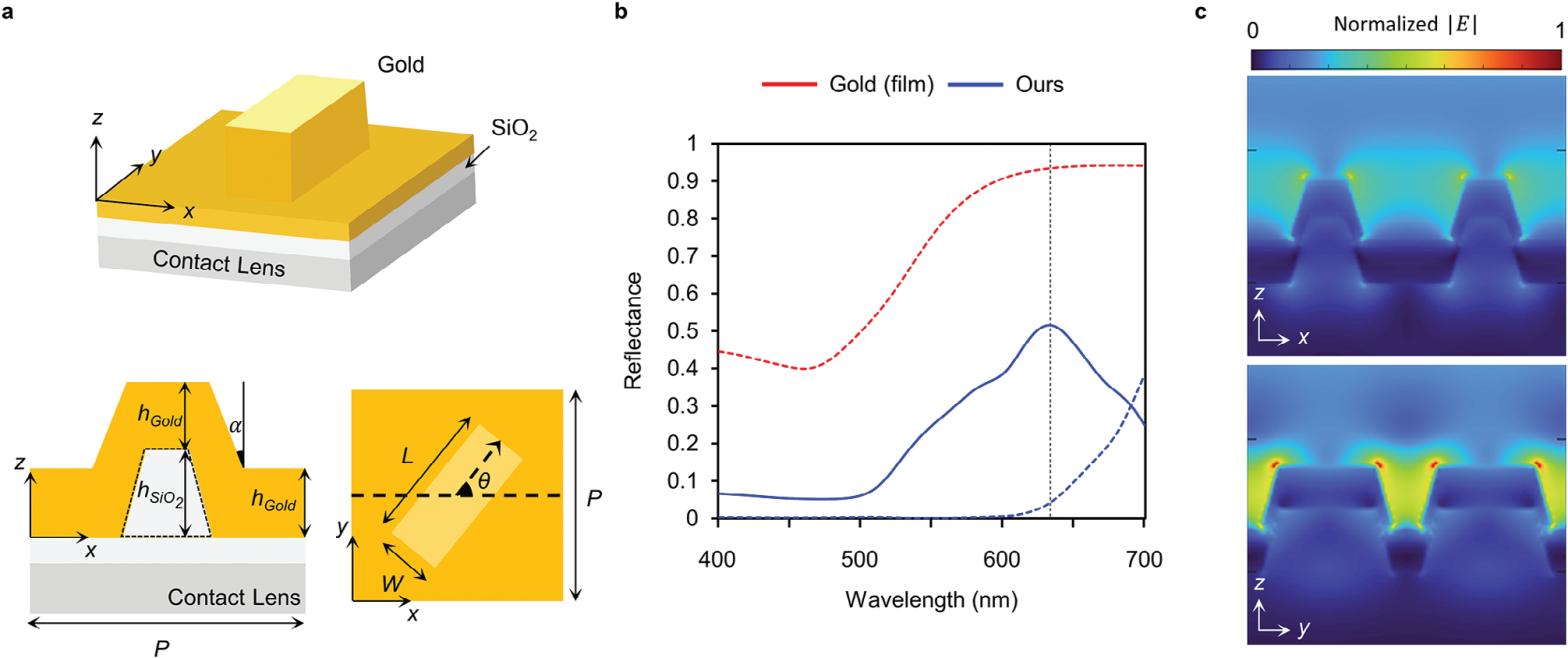
All‐metallic 3D metasurface design a) Schematic of unit 3D meta‐atom. At the top of contact lens, the SiO_2_ capping layer supports the upper 3D gold layer. Geometrical parameters are given as: *h_Gold_* = 80 nm, $h_{SiO_{2}}$ = 120 nm, length *L* = 250 nm, width *W* = 140 nm, periodicity *P* = 300 nm, tapered angle 𝛼 = 15°. The geometrical parameters are identical for every unit cell, except for the in‐plane rotation angle 𝜃. b) Simulated reflectance of gold film (80 nm) and 3D meta‐atom. Solid and dotted line represent helicity‐converted and unconverted reflection, respectively. Vertical line denotes the target wavelength of 637 nm. c) Simulated |*E*| field in *x‐z* and *y‐z* planes under circularly‐polarized light incidence.

A schematic of the metasurface‐embedded contact lens is shown in **Figure** **1**. In the reflection channel, the wavefront manipulated by the metasurface propagates to the far‐field to generate a desired holographic image. For phase‐only computer‐generated hologram (CGH) retrieval, the Gerchberg–Saxton (GS) algorithm, consisting of iterative Fourier transform, was used. To implement the retrieved phase‐only CGH with high efficiency, the unit nanostructures, called meta‐atoms, are designed to be of the same size but with different in‐plane rotation angles, at which phase manipulation is manifested by the Pancharatnam–Berry (PB) phase. Exploiting the PB phase, the reflected wavefront of metasurface can have a spatially varying phase distribution with uniform amplitude. Under incidence of circularly polarized light, the reflected electric field is described as:

(1) $$\frac{r_{l}-r_{s}}{2}e^{\pm i2\theta \left(x,y\right)}\left[\begin{array}{c} 1\\ \pm i \end{array}\right]+\frac{r_{l}+r_{s}}{2}\left[\begin{array}{c} 1\\ \mp i \end{array}\right]$$

where *r_l_* and *r_s_* represent the reflection coefficients along the long and short axes of the meta‐atom which are determined by the geometrical parameters of the meta‐atom, and θ(*x*, *y*) represents the spatially‐varying in‐plane rotation angle (**Figure** **2**a). Here, it should be noted that only helicity‐converted light experiences a spatially varying phase delay, equivalent to twice the rotated angle, where helicity denotes the absolute direction of electric field rotation. Therefore, the reflection coefficients should be designed in a way that increases the absolute value of $\frac{r_{l}-r_{s}}{2}$ to enhance the efficiency of helicity conversion. Preliminarily, silicon dioxide (SiO_2_) which has a similar refractive index to the contact lens is embedded with our metallic unit cell to prevent the unwanted distortion of the periodicity of the meta‐atom induced by swelling and shrinkage of the moisturized contact lens.

We analyzed the geometric configuration of the metallic unit cell to increase conversion efficiency in the reflection channel. Although gold material exhibits high ohmic loss at visible wavelengths, the 80 nm gold film structure could be utilized to achieve a high simulated reflectance (93.5%) at 637 nm (Figure 2b). However, all the reflected light maintains the same helicity as that of the incidence. Leveraging the high reflectance of the all‐metallic layer, our all‐metallic 3D meta‐atoms, by exploiting the anisotropy of rectangular shapes, could achieve a high conversion efficiency in the reflection channel, 51.4% in simulation, with unconverted light lower than 5%. Compared to the widely used metal‐insulator‐metal (MIM) structure, which exploits Fabry–Perot resonance between the top anisotropic gold meta‐atoms and the bottom gold mirror,^[^ ^30^ ^]^ our anisotropic 3D meta‐atoms achieve high conversion efficiency from the waveguide generated in the low‐loss air between meta‐atoms (Figure 2c). With a non‐resonant mechanism of helicity conversion, the ohmic loss of gold material could be alleviated.^[^ ^31^, ^32^ ^]^ It should be noted that the 2D rectangular‐shaped gold embedded in SiO_2_ has much lower conversion efficiency compared to our 3D meta‐atom. Without a background metal layer, the transmitted light increases, and air waveguides are weakly generated, resulting in low conversion efficiency (Figure S1, Supporting Information). Also, the helicity conversion is not strongly affected by the embedded material of the capping layer, which stems from the highly reflective property of our all‐metallic 3D meta‐atoms. As shown in Figure S2a (Supporting Information), the conversion efficiencies remain similar regardless of the material in the dashed region in the side view of Figure 2a.

### 2.2 Fabrication of Metasurface‐Embedded Contact Lenses

**Figure 3:**
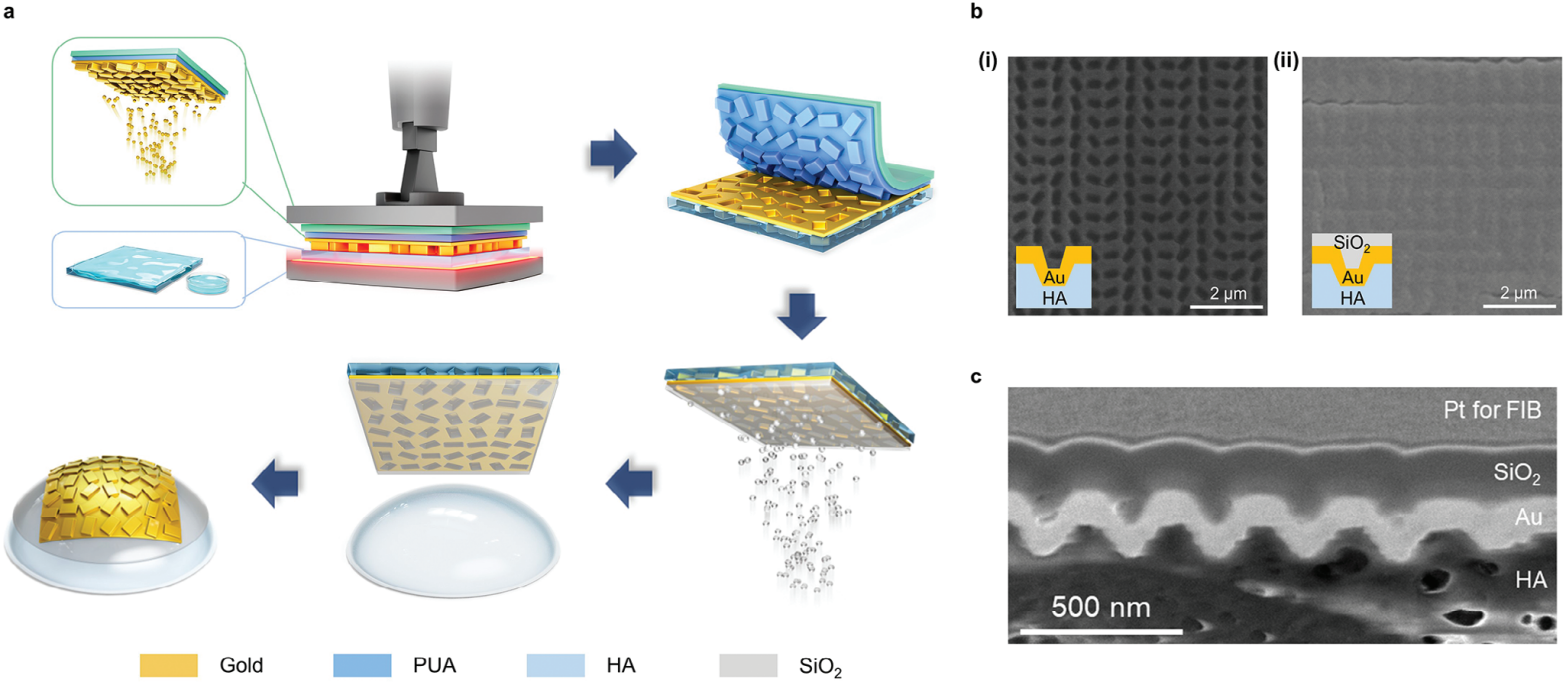
Fabrication of metasurface‐embedded contact lens. a) Schematic of transferring metasurface onto contact lens using biocompatible hyaluronic acid (HA) material and SiO_2_ capping layer. b) Scanning electron microscope (SEM) image and cross‐sectional schematic of the transferred 3D gold metasurface on HA. (i) Before and (ii) after SiO_2_ deposition. c) FIB image of corrugated 3D gold metasurface encapsulated by SiO_2_.

Building upon our previous work,^[^ ^29^ ^]^ we advanced it by implementing a complex 3D nanostructured metasurface on contact lenses using hyaluronic acid (HA). Renowned for its exceptional biocompatibility and water‐retention properties, HA emerged as the prime candidate for the sacrificial layer material in our investigation. This biopolymer, naturally present in the human body, not only offers a high degree of compatibility with ocular tissues but also exhibits remarkable moisture retention capabilities, essential for maintaining ocular hydration and comfort. Leveraging these inherent characteristics of HA, we sought to integrate it into the fabrication process of our metasurface‐enabled contact lenses, ensuring not only the optical functionality of the lenses but also their compatibility with the delicate ocular environment. Additionally, any residual HA is removed during the fabrication process, and HA is naturally found in human tears, ensuring its safety for ocular applications. As shown in **Figure** **3**a, the process involved the deposition of gold on the polyurethane acrylate (PUA) soft mold, replicated from the silicon master metasurface and transferring the corrugated 3D gold onto a hydrated HA film. To facilitate the transfer, the HA film was sufficiently hydrated to reach a deformable state followed by the application of heat and pressure to embed the 3D gold metasurface into the HA film. From the obtained scanning electron microscope (SEM) images, the backside of the corrugated 3D gold metasurface is observed to have a hole‐like shape (Figure 3b (i)). Subsequently, a SiO_2_ capping layer was introduced to ensure the structural stability of the corrugated 3D nanostructures, and the corresponding SEM image is shown in Figure 3b (ii). The cross‐sectional image analyzed by a focused‐ion beam (FIB) shows that the corrugated 3D Au metasurface is fully embedded in the HA film and densely covered by the SiO_2_ deposited on it. The SiO_2_ capping layer, situated between the contact lens material and the gold patterns, prevents direct contact with the eye, thereby minimizing any potential risk of adverse effects. Upon orienting the metasurface with the nanostructures on the HA template, toward the contact lens, the HA initiates dissolution in response to the inherent moisture within the contact lens. This dissolution process facilitates the placement of the metasurface onto the contact lens, facilitated by the transformative gel‐like state assumed by the HA. The residual HA was subsequently dissolved in water, resulting in the final metasurface‐embedded contact lens. The cross‐sectional image of the metasurface‐embedded contact lens revealed a seamless integration between the contact lens and the metasurface, with no observable air gap and structural distortion facilitated by the SiO_2_ capping layer. Additionally, the presence of residual HA was nearly negligible by FIB analysis (Figure S3, Supporting Information).

### 2.3 Metahologram Demonstration Through Metasurface‐Embedded Contact Lenses

**Figure 4:**
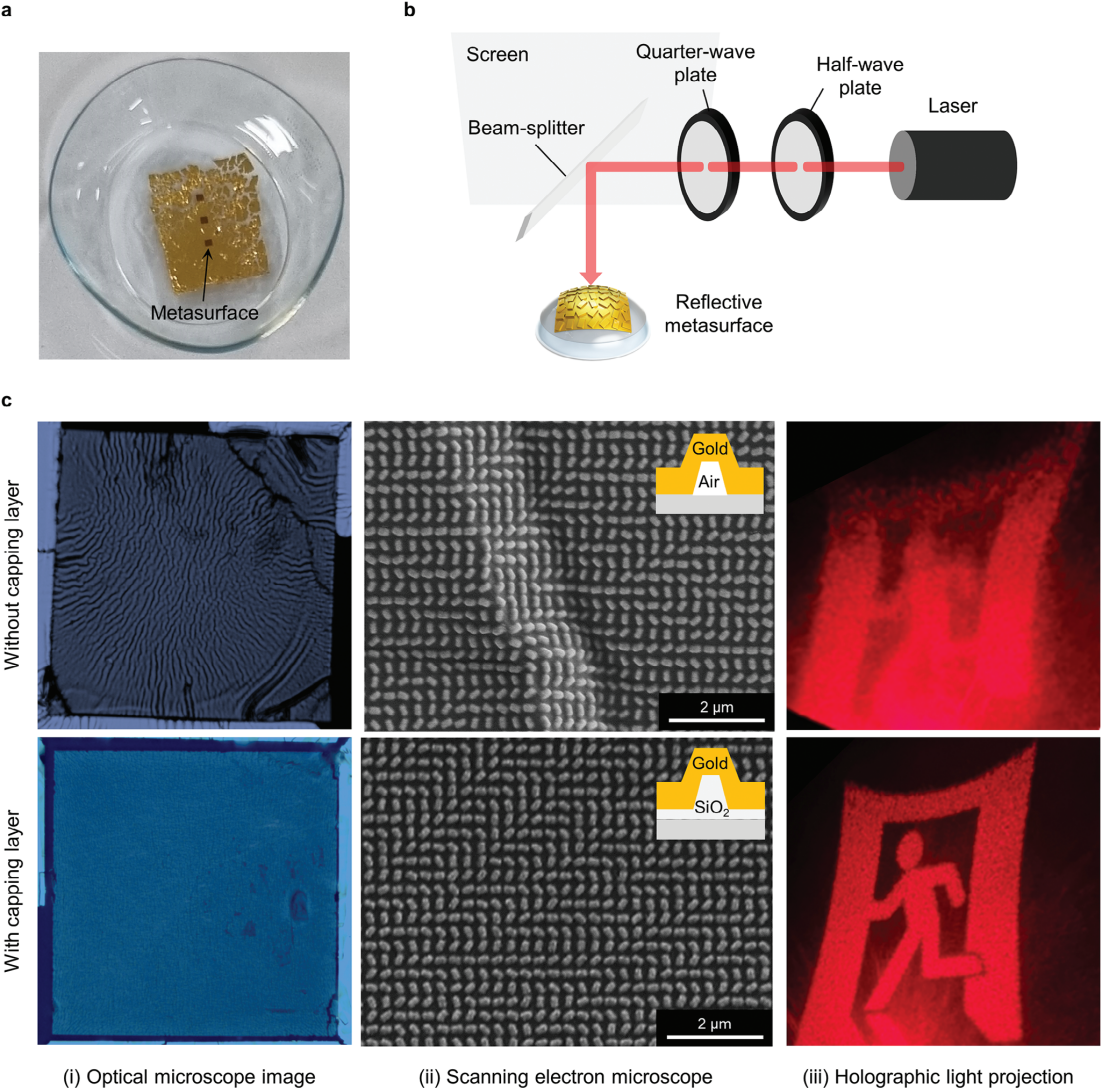
Experimental demonstration of metasurface‐embedded contact lens a) Transferred all‐metallic 3D metasurface onto the surface of contact lenses. b) Optical setup for holographic light projection paired with illumination module for the circularly‐polarized light incidence. c) The effect of the SiO_2_ capping layer on the stability of pattern transfer. Without capping layer, the wrinkle by sudden expansion of HA upon contact with moisturized contact lens is observed in both (i) OM and (ii) SEM image. (iii) Reflected holographic images from metasurface‐embedded contact lens.

The metasurface‐embedded contact lenses (**Figure** **4**a) underwent a visual validation process, involving laser irradiation at a wavelength of 637 nm to generate a meta‐hologram. The half‐wave plate and quarter‐wave plate were incorporated for the circularly polarized light incidence (Figure 4b). The fabricated metasurface‐embedded contact lens was placed on a glass slide, and the reflected image was quantified. The holographic images obtained from two different samples, the one without SiO_2_ capping layer and the other with SiO_2_ capping layer deposition, are presented in Figure 4c. The absence of a SiO_2_ capping layer results in poor structural stability, as shown by the wrinkle pattern on an optical microscope (OM), and scanning electron microscope (SEM) image (Figure 4c). Without the SiO_2_ capping layer, unwanted wrinkles are observed over the entire pattern of the metasurface, arising from swelling and shrinkage during the transfer to the moisturized contact lenses, resulting in a degraded holographic image. Leveraging structural stability induced by the SiO_2_ capping layer, the metasurface pattern on the HA film is stably transferred to the contact lens without further distortion and therefore the reconstructed holographic image could match the original target image without degradation of detailed features. The helicity‐conversion efficiency, which contributes to the holographic image generation, is measured at 25.7% in the experiment. The discrepancy is attributed to the periodic boundary condition used to accelerate the FDTD simulation, which is inconsistent with the fabricated metasurface composed of the highly non‐uniform rotation angle of meta‐atoms and therefore the helicity conversion induced by low loss air waveguide between meta‐atoms is degraded compared to the periodic simulation. Furthermore, since contact lenses are constantly exposed to a moist environment due to tear production, it is crucial to assess their structural and performance stability after prolonged exposure to wet conditions and under varying relative humidity (RH) levels. In both characterizations, we revealed that the performance of the metahologram remained stable without degradation (Note S1, Supporting Information).

## 3 Conclusion

In conclusion, this study has successfully demonstrated the integration of a metasurface with a 3D nanostructure into a contact lens through a biocompatible process. The developed metasurface, comprising all‐metallic 3D meta‐atoms, enhanced by an anisotropic and highly reflective background metal layer with a rectangular structure, exhibited a conversion efficiency of 51.4% in simulation and 25.7% in experimentation at the reflective channel. The incorporation of the 3D meta‐atom into contact lenses utilized HA as a water‐soluble and biocompatible template. By controlling ambient humidity to induce deformability in the HA template, the application of heat and pressure facilitated the embedding of the 3D Au nanostructure into the HA template. The introduction of SiO_2_ as a capping layer played a crucial role in minimizing undesired defects during the integration process and served as a waveguide, leveraging the low‐loss air between the meta‐atoms. This strategic addition significantly improved both process stability and overall performance. Furthermore, the visualized holographic light projection confirmed the feasibility of creating smart contact lenses with a compact form factor. Our work introduces several crucial innovations, including an advanced biocompatible process, enhanced structural stability, and high hologram efficiency, marking a significant advancement in the field. The successful integration of metasurface technology into contact lenses opens new avenues for advancing visual experiences in AR displays and enhancing human‐computer interaction through the incorporation of diverse optical components. While these findings are promising for compact near‐eye displays (NEDs), there are still some aspects that can be enhanced, taking into account the properties of human vision, such as optical see‐through, field‐of‐view, and eyebox (Note S2, Supporting Information). The findings of this study contribute to the evolving landscape of wearable technologies, particularly in the context of ophthalmic applications, with potential implications for broader applications in AR and related fields.

## 4 Experimental Section

### Metasurface Fabrication

The master metasurface mold was patterned on a silicon (Si) substrate. The designed metasurface pattern was transferred to a positive photoresist (950 PMMA A2, MicroChem) through electron‐beam lithography (ELS‐7800, ELIONIX). After developing exposed patterns using MIBK/IPA 1:3 developer, 40 nm‐thick chromium (Cr) was deposited using an electron beam evaporator (KVE‐ENS4004, KVT), followed by a subsequent lift‐off. Using the Cr patterns as an etching mask, the metasurface pattern was transferred to the Si layer by a dry etching process (DMS, silicon/metal hybrid etcher). Finally, the remaining Cr mask was removed using Cr etchant (CR‐7).

### Replication of Metasurface

Si master by e‐beam lithography was replicated using UV‐curable polyurethane acrylate (PUA) resin (311‐RM, Munuta Tech. Co., Ltd., Korea). The resin was dropped onto the Si master, which had been covered with polyethylene terephthalate film as a support. Pressure was applied to remove the trapped air, and the resin was filled into the meta‐atoms using a roller. Afterward, the template was UV cured twice for the 90s each. Finally, Au was deposited on the detached PUA mold using an e‐beam evaporator at a rate of 1.5^−2^Ås^−1^ under high vacuum.

### Hydrated HA Film

A suspension of HA powder (HA‐TLM 20−40 (200−400 kDa); Bloomage Biotechnology Corporation Ltd., China) in deionized water was stirred at 550 rpm for 4 h at room temperature (23 °C) to prepare a 1 wt.% solution, which was then poured into a Petri dish fixed with a 2″ bare Si wafer. The dish was placed in a vacuum chamber for 10 min to remove air bubbles and dried at 50 °C in a convection oven for 1 day. After the removal of the Si wafer, the sample was fixed on a 4″ Si wafer and treated with O_2_ plasma for 30 s. For sufficient hydration, the HA film was placed in a closed container next to a 2″ Petri dish filled with hot water, which ensured that at least 80% humidity was maintained inside the container.

### Nanotransfer Printing on HA Film

The PUA mold, containing the deposited gold, and the hydrated HA film was stacked and attached together. The mold was subsequently pressurized for 5 min at ≈5 bar and 100 °C, cooled, and then separated from the HA film.

### Nanotransfer Printing on Contact Lens

A soft contact lens (1‐DAY ACUVUE TruEye, Johnson & Johnson Vision Care, Inc., Jacksonville, FL) was placed on a sliding glass. After placing the HA template with the embedded metasurface on the contact lens, a container of hot water was placed inside the square Petri dish to help dissolve the hyaluronic acid. Once the steam had sufficiently dissolved the hyaluronic acid, it was allowed to dry, so that the metasurface could be secured to the contact lens. Finally, to remove any remaining hyaluronic acid, the lenses were fully submerged in the container of water.

### Metasurface Simulation

The finite‐difference‐time‐domain (FDTD) from Lumerical was used to characterize the conversion efficiency of the metasurface. The periodic boundary conditions are used in *x*‐ and *y*‐directions, and the perfectly matched layers are used in *z*‐directions. Under circularly polarized light incidence, the electric field reflected from the metasurface was decomposed into *x*‐ and *y*‐direction to calculate the efficiency of polarization‐converted and unconverted light.

### Experimental Setup

The illumination module consists of a linearly‐polarized laser at a wavelength of 637 nm (MDL‐III‐637), a half‐wave plate (AHWP10M‐600) and a quarter‐wave plate (AQWP05M‐600). The half‐ and quarter‐wave plates are used to alter the linearly‐polarized laser light into circularly‐polarized light. In addition, the performance of holographic images under varying relative humidity (RH) was characterized using a homemade chamber equipped with a humidifier and a humidity meter (Lutron, HT‐3007SD). Humidity under ambient RH was achieved using silica gel. Figure S5 (Supporting Information) shows the detailed experimental setup.

## Conflict of Interest

The authors declare no conflict of interest.

## Author Contributions

J.K. and G.K. contributed equally to this work. J.R., I.P., and J.‐H.J. conceived the idea. J.K. performed nanotransfer printing to fabricate metasurface‐embedded contact lenses. G.K. designed and simulated the metasurface‐embedded contact lenses. I.K. fabricated the metasurface master mold. G.K. implemented the experimental setup. S.‐H.H., S.J., J.A., Y.J., and J.‐H.H. provided advices on nanotransfer printing. H.H. assisted on the metasurface simulation. All authors participated in discussions and contributed to writing the manuscript. J.R., I.P., and J.‐H.J. guided all aspects of the work.

## Supporting information

Supporting Information

## Data Availability

The data that support the findings of this study are available from the corresponding author upon reasonable request.
