# Supplementary material for: Metasurface‐Embedded Contact Lenses for Holographic Light Projection
Source: Adv Sci (Weinh). 2024 Aug 9;11(38):2407045. doi: 10.1002/advs.202407045 (PMC11481215; doi:10.1002/advs.202407045)
Supplement: Supplementary file 1 — Supporting Information [file ADVS-11-2407045-s001.docx]

**Supporting Information**

**Metasurface-Embedded Contact Lenses for Holographic Light Projection**

Jiwoo Ko^1,2#^, Gyeongtae Kim^3#^, Inki Kim^3,4,5^, Soon Hyoung Hwang^2^, Sohee Jeon^2^, Junseong Ahn^6^, Yongrok Jeong^7^, Ji-Hwan Ha^1,2^, Hyeonsu Heo^3^, Jun-Ho Jeong ^2*^, Inkyu Park ^1*^, Junsuk Rho^3,8,9,10,11*^

^1^Department of Mechanical Engineering, Korea Advanced Institute of Science and Technology (KAIST), Daejeon 34141, South Korea.

^2^Department of Nano Manufacturing Technology, Korea Institute of Machinery and Materials (KIMM), Daejeon 34103, South Korea

^3^Department of Mechanical Engineering, Pohang University of Science and Technology (POSTECH), Pohang 37673, Republic of Korea

^4^Department of Biophysics, Institute of Quantum Biophysics, Sungkyunkwan University, Suwon 16419, Republic of Korea

^5^Department of Intelligent Precision Healthcare Convergence, Sungkyunkwan University, Suwon 16419, Republic of Korea

^6^Department of Electro-Mechanical Systems Engineering, Korea University, Sejong 30019 Republic of Korea

^7^Radioisotope Research Division, Korea Atomic Energy Institute, Daejeon 34057, Republic of Korea

^8^Department of Chemical Engineering, Pohang University of Science and Technology (POSTECH), Pohang 37673, Republic of Korea

^9^Department of Electrical Engineering, Pohang University of Science and Technology (POSTECH), Pohang 37673, Republic of Korea

^10^POSCO-POSTECH-RIST Convergence Research Center for Flat Optics and Metaphotonics, Pohang 37673, Republic of Korea

^11^National Institute of Nanomaterials Technology (NINT), Pohang 37673, Republic of Korea

*Corresponding author. E-mail: [jsrho@postech.ac.kr](mailto:jsrho@postech.ac.kr), [inkyu@kaist.ac.kr](mailto:inkyu@kaist.ac.kr), [jhjeong@kimm.re.kr](mailto:jhjeong@kimm.re.kr)

^#^These authors contributed equally to this work.

**
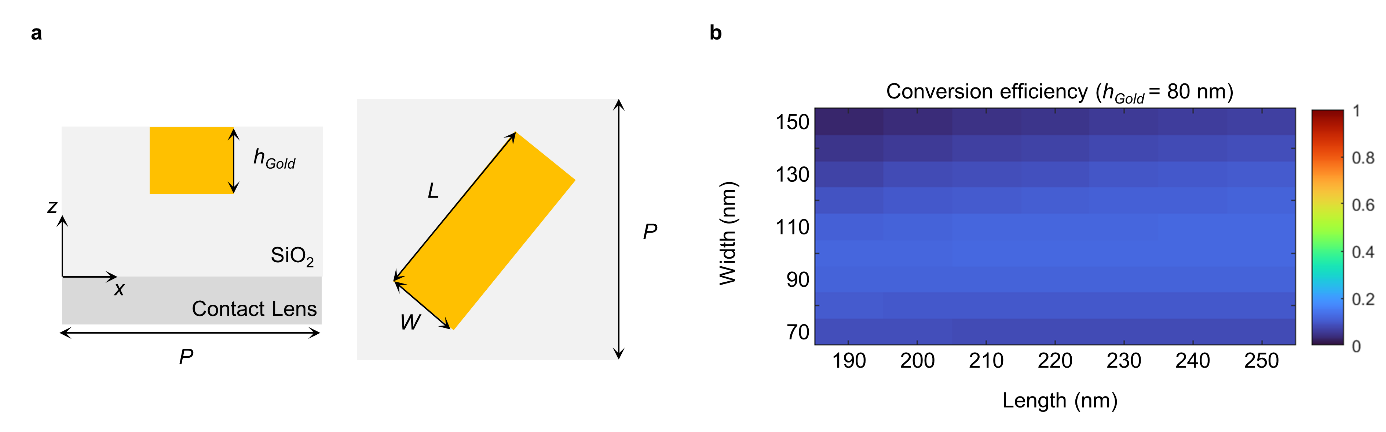
**

**Figure S1.** 2D rectangular-shaped gold metasurface. (a) Schematic of a rectangular-shaped 2D gold meta-atom embedded in SiO_2_. The refractive indices of the capping layer and the contact lens are the same. (b) Simulated conversion efficiencies of the rectangular-shaped gold in the reflection channel by varying length and width of gold structures.


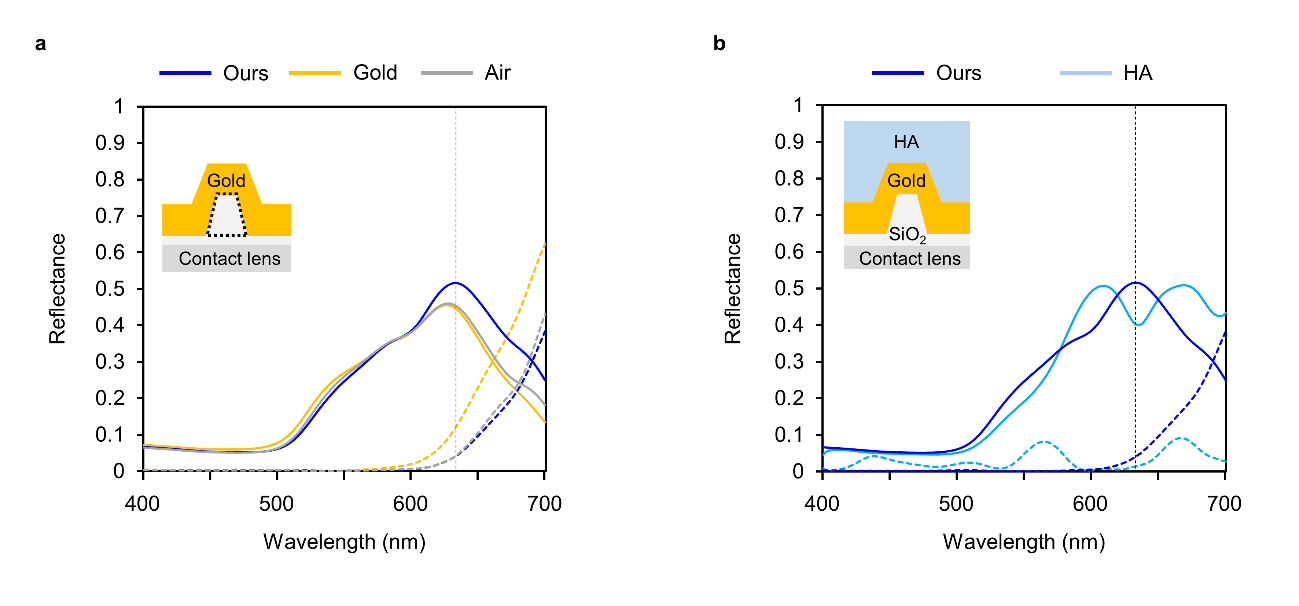


**Figure S2**. Effect of capping layer material and residual HA. Simulated reflectance of our 3D meta-atom in main text is plotted in both (a) and (b). Solid and dotted plots represent helicity-converted and unconverted reflection, respectively. Vertical dotted line denotes the target wavelength of 637 nm. (**a**) Simulated reflectance by varying the capping layer material in the dashed area of inset. (**b**) Simulated reflectance in the presence of residual HA.


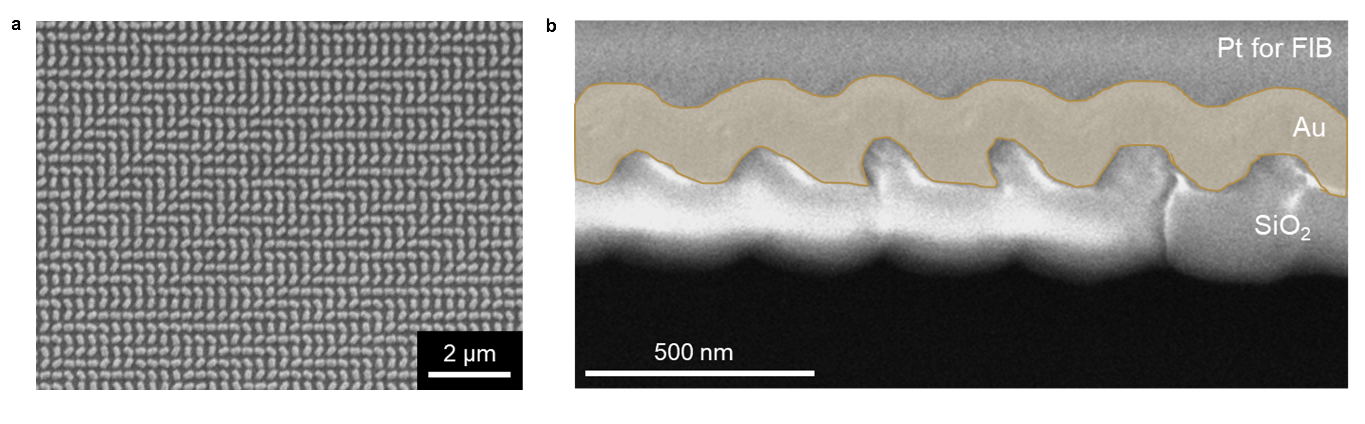


**Figure S3.** Metasurface-embedded contact lens. **(a)** Surface image by SEM analysis and **(b)** cross-sectional image by FIB analysis.

**Supplementary Note 1: Performance Evaluation in Humid Environment**


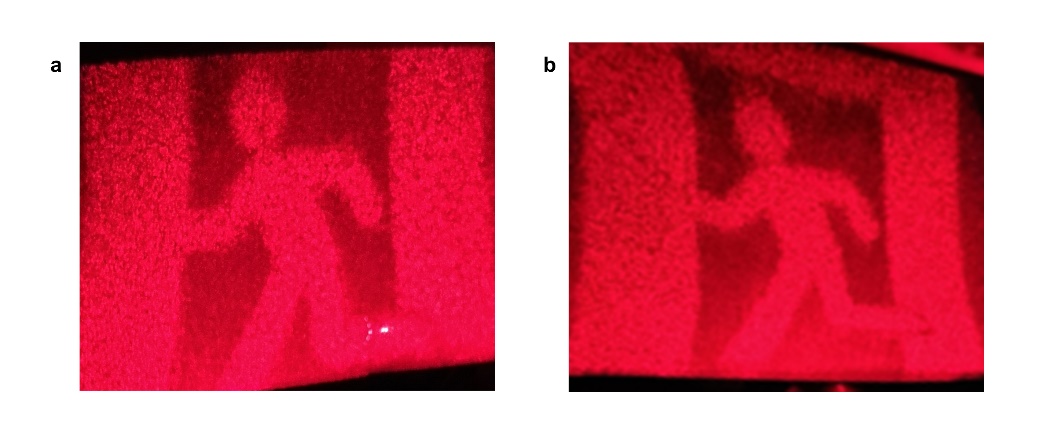


**Figure S4**. Metasurface-embedded contact lens under wet conditions. Holographic image (**a**) before and (**b**) after submerge. The quality of the holographic image preserves even after immersion into the water.


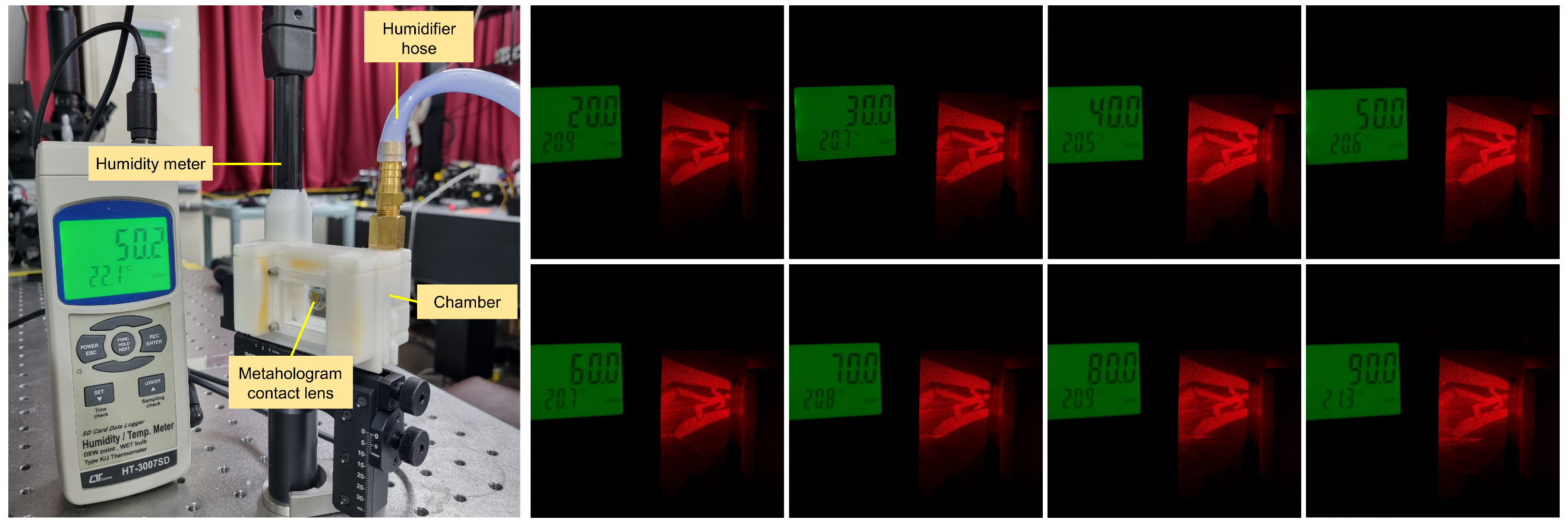


**Figure S5.** The performance of holographic light projection under varying relative humidity (RH) from 20% to 90%. A metasurface-embedded contact lens is mounted inside a custom-made chamber equipped with a humidifier and humidity meter. The reflected holographic image is observed through a window, and the performance of holographic images remained stable across different RH levels.

**Supplementary Note 2: Near-eye Displays Analysis**

An augmented reality (AR) display system includes a near-eye display paired with an imaging system designed to address the limited accommodation of the human eye-lens. Simultaneously, the virtual image across different depths should be remained in-focus while maintaining optical see-through over real-scene. Leveraging a pinhole imaging system within AR displays, an all-in-focus virtual image can be consistently delivered onto the retina without the need for additional focal-length tuning mechanisms^1-3^ (Fig. S6). As long as the display is located within the viewing cone with its vertex at the center of the pinhole, the in-focused virtual image can be seen regardless of the eye-lens’s focused distance. However, this method encounters inherent limitations: a narrow field-of-view (FoV) due to the distance between the pinhole and the pupil, and a limited eyebox because of a single viewing cone with a fixed vertex at the pinhole location. The eyebox is the area within which the pupil must stay to fully view the augmented displays. To overcome these challenges, pinhole arrays have been introduced, but they employ multiple viewing cones, and hence resulting in stitched images with significant artifacts along the boundaries^4^.


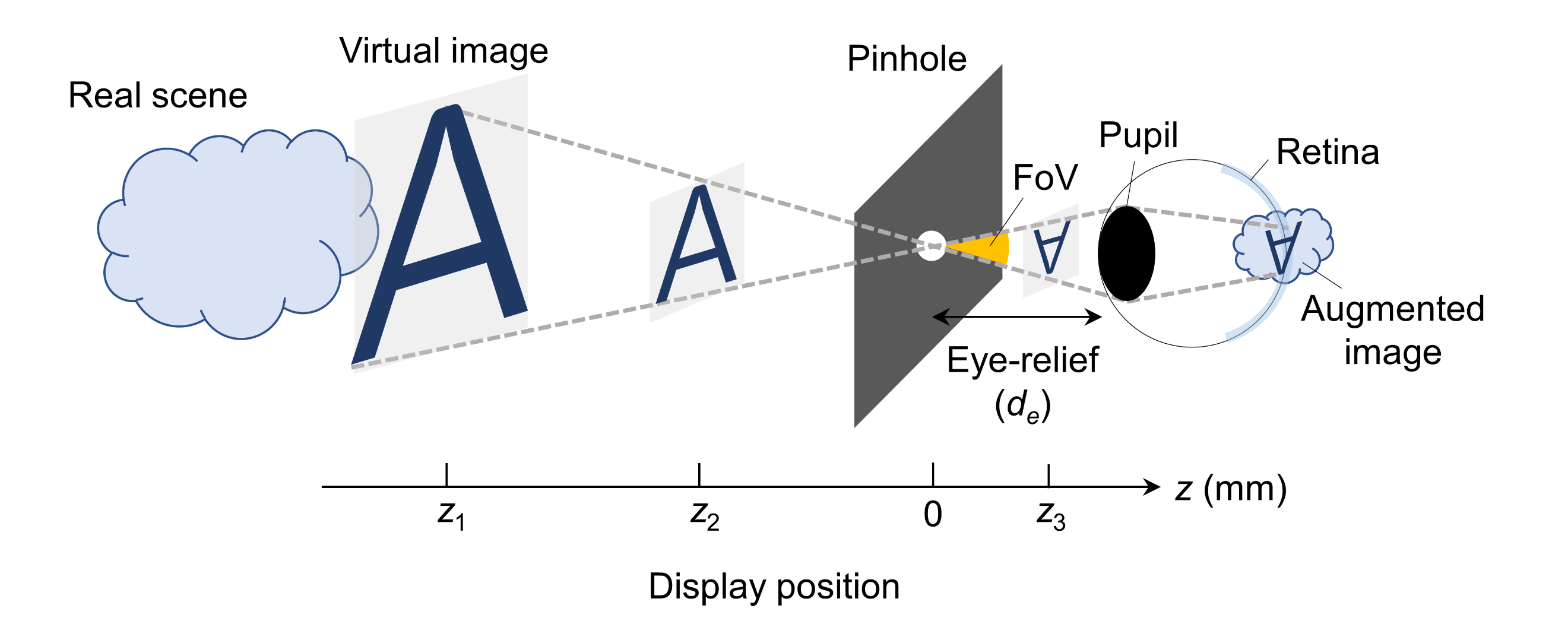


**Figure S6.** All-in-focus near-eye-display with pinhole imaging system. The virtual image augmented on the real-scene consistently remains in-focus across various distances of real-scene objects. Specifically, the metahologram positioned at $z\geq0$, paired with backlight coherent laser source, projects the virtual image onto retina while maintaining optical see-through with the small footprint of the metahologram.

In this work, the proposed metahologram operates on the reflection channel but can be readily converted to a transmissive metahologram by designing meta-atoms with high conversion efficiency through modulating transmission coefficients $t_{l}$ and $t_{s}$^5, 6^. If re-designed as a transmissive metasurface, our metahologram-embedded contact lenses could surpass the FoV of the extant pinhole-aided all-in-focus AR displays, which is achieved by eliminating the need for eye-relief distance by directly transferring the metahologram onto the pupil and exploiting the subwavelength lattice period of meta-atoms which allows for a large diffraction angle^7, 8^. Consequently, a single metahologram can provide wide FoV augmented displays without the need for stitching sub-images.

Regarding the eyebox, the metasurface must be accurately aligned with the center of the pupil to provide an uncropped view of the full virtual display. Most contact lenses feature a mark that distinguishes the front from the back, similar to alignment marks used in photolithography processes. These marks can ensure precise alignment when implementing metasurfaces onto contact lenses. Additionally, commercial contact lenses are designed to fit securely over the cornea, an elliptical structure of the eye. Due to their curvature, size, and flexible material properties, contact lenses exhibit minimal movement once worn. Therefore, after integrating the metasurface onto the contact lenses with precise alignment, the eyebox is maintained regardless of the eye’s position and viewing direction. Furthermore, the optical see-through of the real-scene could be preserved, as the size of metaholograms (300μm^2^) account for 0.25% of the pupil size, minimally occluding the real-scene view.

**Reference**

1. G. Palermo, A. Lininger, A. Guglielmelli, L. Ricciardi, G. Nicoletta, A. D. Luca, J.-S. Park, S. W. D. Lim, M. L. Meretska, F. Capasso, G. Strangi, *ACS Nano* 2022, **16**, 10, 16539–16548.
2. M. Bosch, M. R. Shcherbakov, K. Won, H.-S. Lee, Y. Kim, G. Shvets, *Nano Lett*. 2021, **21**, 9, 3849–3856.
3. T. Badloe, I. Kim, Y. Kim, J. Kim, J. Rho, *Adv. Sci.* 2021, **8**, 2102646.
4. A. Maimone, D. Lanman, K. Rathinavel, K. Keller, D. Luebke, H. Fuchs, *ACM Trans. Graph.* 2014, **33**, 89.
5. J. Kim, D. K. Oh, H. Kim, G. Yoon, C. Jung, J. Kim, T. Badloe, H. Kang, S. Kim, Y. Yang, J. Lee, B. Ko, J. G. Ok, J. Rho, *Laser Photon. Rev.* 2022, **16**, 2200098.
6. I. Kim, J. Jang, G. Kim, J. Lee, T. Badloe, J. Mun, J. Rho, *Nat. Commun*. 2021, **12**, 3614.
7. G. Kim, Y. Kim, J. Yun, S. W. Moon, S. Kim, J. Kim, J. Park, T. Badloe, I. Kim, J. Rho, *Nat. Commun.* 2022, **13**, 5920.
8. E. Choi, G. Kim, J. Yun, Y. Jeon, J. Rho, S.-H. Baek, *Nat. Photon.* 2024.
